# P29 A nurse-run, pharmacist-led outpatient penicillin allergy de-label clinic in Cornwall

**DOI:** 10.1093/jacamr/dlaf118.036

**Published:** 2025-07-14

**Authors:** Neil Powell, Daniel Hearsey, Tamsyn Lewis, Marie Thomas, Helen Winn, Amanda Pritchard, Sharon Clare, Jane Williams, Sophie Weeks, Amanda Mcdonald

**Affiliations:** Royal Cornwall Hospital Trust, Truro, Cornwall, TR5 0QB, UK; Royal Cornwall Hospital Trust, Truro, Cornwall, TR5 0QB, UK; Royal Cornwall Hospital Trust, Truro, Cornwall, TR5 0QB, UK; Royal Cornwall Hospital Trust, Truro, Cornwall, TR5 0QB, UK; Royal Cornwall Hospital Trust, Truro, Cornwall, TR5 0QB, UK; Royal Cornwall Hospital Trust, Truro, Cornwall, TR5 0QB, UK; Royal Cornwall Hospital Trust, Truro, Cornwall, TR5 0QB, UK; Royal Cornwall Hospital Trust, Truro, Cornwall, TR5 0QB, UK; Royal Cornwall Hospital Trust, Truro, Cornwall, TR5 0QB, UK; Royal Cornwall Hospital Trust, Truro, Cornwall, TR5 0QB, UK

## Abstract

**Background:**

Penicillin allergy (penA) records are common, but 95% of patients with penA records are able to take penicillin antibiotics after formal allergy testing.^1^ PenA records are associated with broad spectrum antibiotic prescribing and negative patient and health-system outcomes which makes removal of incorrect penA records (penicillin allergy de-labelling; PADL) an antimicrobial stewardship and patient safety priority.^2^ The paucity of allergy specialists world-wide has led to non-allergy healthcare worker delivered PADL. Nurses in HK have shown they can safely deliver a direct oral challenge (DOC) of penicillin for low-risk patients in the outpatient setting, supervised by allergists.^3^ We set up nurse run adult low risk PADL outpatient clinic, supervised by an antibiotic pharmacist.

**Methods:**

PADL guidelines written and approved off by the hospital’s Medicines Practice Committee. PADL training to nurses was provided by the supervising antibiotic pharmacist. The clinic started accepting electronic referrals from the hospital outpatient clinics and one GP surgery in Cornwall 27th December 2024. Telephone triage of patients started 6th January 2025. Nurse telephone triage includes taking a penicillin allergy focused history, risk assessment of the penA history and determination of PADL method, if appropriate. Histories are checked by the supervising pharmacist as a safety check before the patient is invited for a direct oral penicillin challenge test. Ten day post-test follow-up telephone call is made by a member of the nursing team to enquire about any potential side effects from the DOC and the GP emailed with a clinic letter. The first outpatient clinic was 7 February 2025.

**Results:**

Between 27 December 2024 and 4 April 2025 we have received 129 referrals, of which 114 were successfully contacted and a penA focused history taken over the telephone. Of these, 36/114 (31.6%) had a high-risk penicillin allergy history and 3/114 (2.6%) met other exclusion criteria and 3 patients declined attending the DOC clinic. All were excluded. 72/114 (63.1%) were categorized as low risk. Of these, 58/72 (80.6%) agreed to attend clinic for DOC and 10/72 (13.9%) were de-labelled on history alone. To date, 42 patients have received their DOC in the outpatient clinic of which 31 have had their 10 day follow-up telephone call. Of those followed up, 28/31 (90.3%) were successfully de-labelled and 3/31 (9.6%) retained their penA record due to experiencing potential side effects from the DOC; one patient experienced nausea and light-headedness on the way home, one reported a rash day 5 post DOC, and one reported a rash on Day 3 post DOC.

**Conclusions:**

Non-allergy nurse delivered PADL in the outpatient setting is safe and effective at removing low risk penicillin allergy records.
